# Chromatin and gene regulation in archaea

**DOI:** 10.1111/mmi.15302

**Published:** 2024-08-03

**Authors:** Fabian Blombach, Finn Werner

**Affiliations:** ^1^ Division of Biosciences, RNAP Laboratory, Institute of Structural and Molecular Biology (ISMB) University College London London UK

**Keywords:** archaea, chromatin, histones, transcription

## Abstract

The chromatinisation of DNA by nucleoid‐associated proteins (NAPs) in archaea ‘formats’ the genome structure in profound ways, revealing both striking differences and analogies to eukaryotic chromatin. However, the extent to which archaeal NAPs actively regulate gene expression remains poorly understood. The dawn of quantitative chromatin mapping techniques and first NAP‐specific occupancy profiles in different archaea promise a more accurate view. A picture emerges where in diverse archaea with very different NAP repertoires chromatin maintains access to regulatory motifs including the gene promoter independently of transcription activity. Our re‐analysis of genome‐wide occupancy data of the crenarchaeal NAP Cren7 shows that these chromatin‐free regions are flanked by increased Cren7 binding across the transcription start site. While bacterial NAPs often form heterochromatin‐like regions across islands with xenogeneic genes that are transcriptionally silenced, there is little evidence for similar structures in archaea and data from *Haloferax* show that the promoters of xenogeneic genes remain accessible. Local changes in chromatinisation causing wide‐ranging effects on transcription restricted to one chromosomal interaction domain (CID) in *Saccharolobus islandicus* hint at a higher‐order level of organisation between chromatin and transcription. The emerging challenge is to integrate results obtained at microscale and macroscale, reconciling molecular structure and function with dynamic genome‐wide chromatin landscapes.

## Graphical abstract

Recent data from diverse archaea with different repertoires of nucleoid‐associated proteins show a common genome‐wide chromatin organisation where chromatin maintains access to gene promoters independently of transcription activity.

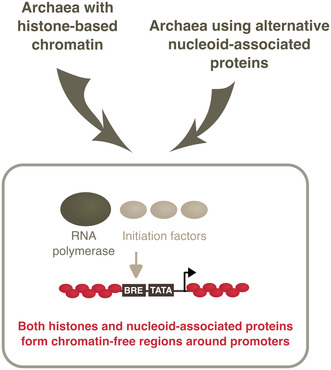

## 1 INTRODUCTION

The control of gene expression links genotype to phenotype of the cell and understanding this process is important for both fundamental and translational research. A plethora of factors need to be considered to rationalise the flow of biological information from genome via transcriptome to proteome. Much research has been focussed on how gene‐specific transcription factors interact with promoter DNA motifs and up‐ or down regulate genes, but recently more complex phenomena that operate at genome‐scale have emerged as important regulatory mechanisms—these are the new frontiers of the field.

Firstly, the two principal processes of gene expression, transcription and translation, are tightly coordinated in all cells. It is likely that in some archaea, as in many bacteria, RNA polymerases (RNAP) and the lead ribosome can physically interact, transcription and translation are physically coupled, which provides an effective means for the translation apparatus to provide direct feedback to the transcription machinery, thus allowing RNAP to ‘sense’ the metabolic state of the cell (Weixlbaumer et al., 2021).

**FIGURE 1:**
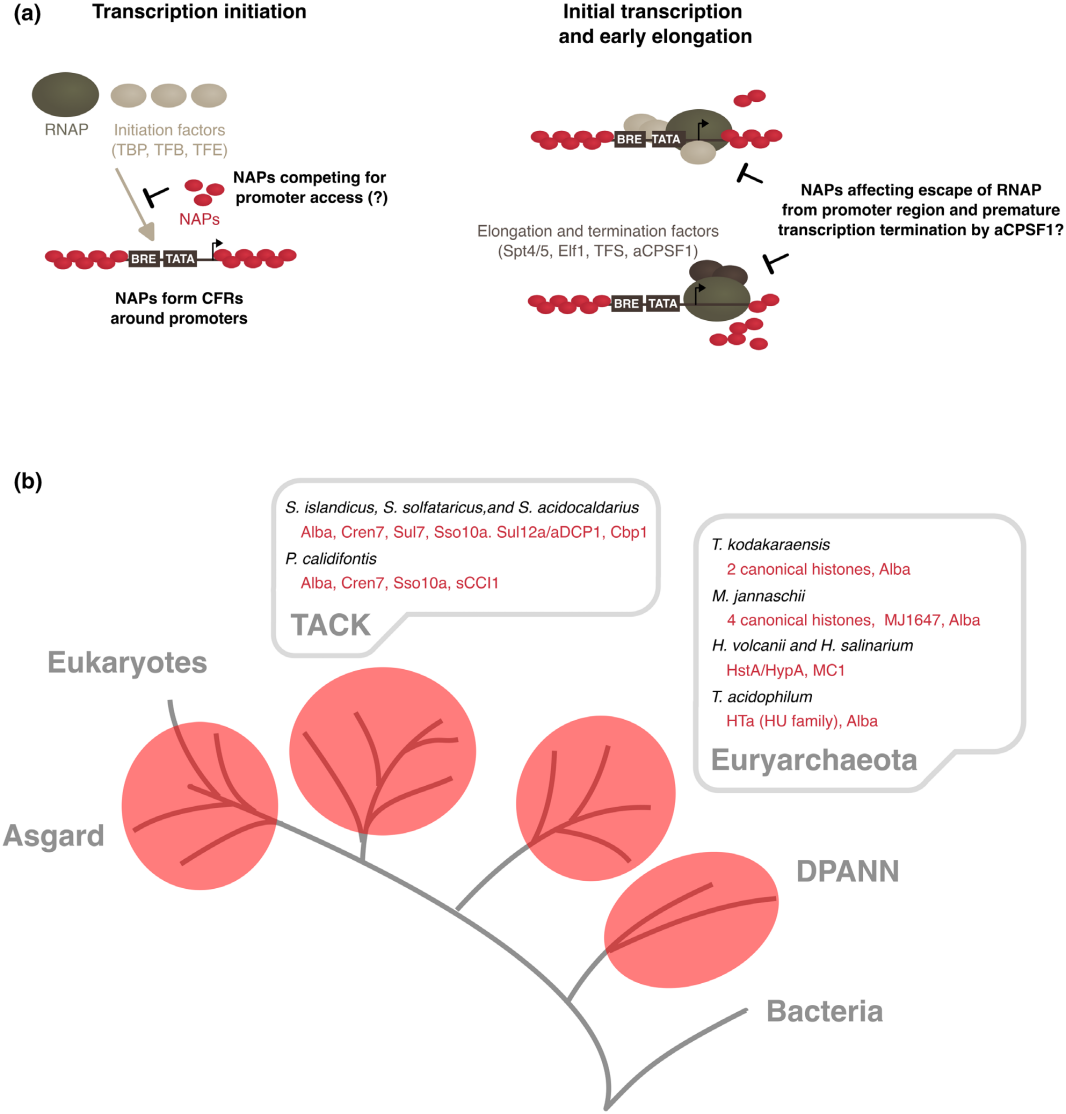
Chromatin‐transcription machinery interactions at promoters. (a) Chromatinisation of DNA and its transcription are two processes affecting each other in multiple, often bidirectional ways. Schematic depicting potential interactions how chromatin could regulate transcription in archaea. Thus far, only the existence of CFRs at promoters has been documented in many archaea, but their role in transcription initiation and RNAP escape remains to be determined. (b) Overview of well‐established archaeal model systems to study the interplay between chromatin and transcription discussed in this review. Experiments have been so far limited to two branches of Archaea: Histone‐chromatinised euryarchaeota and histone‐free species within the crenarchaeota. The NAP repertoire these species is indicated with NAPs discussed highlighted in bold. Note that the histone proteins HstA and HypA show low abundancy and are not considered as proper chromatin proteins.

Secondly, the level of DNA chromatinisation influences gene expression. Arguing from first principles, the chromatinisation of genes can modulate transcription during at least two stages of the transcription cycle, during initiation and elongation. Firstly, NAPs can chromatinise the promoter region blocking access for transcription factors and RNAPs (Figure 1a). This is a key step in transcription regulation in eukaryotes (Ferrie et al., 2022). Chromatinisation also silences cryptic intragenic promoters in *Escherichia coli* (Singh et al., 2014). Second, chromatinisation of intragenic regions of the transcription unit can affect transcription elongation by reducing the average elongation rate of RNA polymerase and by triggering the RNA polymerase to pause, backtrack or even stall (Izban & Luse, 1991; Figure 1a). If NAPs form bridged chromatin filaments as in the case of bacterial H‐NS, the torsional constraints can lead to entrapment of RNA polymerase (Kotlajich et al., 2015). While pausing per se does not alter transcription output in steady state, it can lead to premature termination including utilisation of alternative termination sites. Vice versa, active transcription can alter the chromatinisation of the gene, either directly by the displacement of NAPs from DNA by transcription elongation complexes or indirectly, over longer distances, by generating DNA supercoiling that invades and destabilises NAP filaments, as recently shown in *Salmonella* (Figueroa‐Bossi et al., 2024). Due to this two‐way relationship, it is often difficult to establish causal relationships between chromatin and gene activity based on global NAP occupancy‐ and transcriptome data, both of which represent the steady‐state outcome of the two processes.

## 2 ARCHAEAL NAPs—A BRIEF OVERVIEW

Chromosomes are a heterogenous assembly of nucleic acids and proteins in all domains of life, prokaryotes which are comprised of archaea and bacteria, as well as eukaryotes. In eukaryotes, the canonical octameric histone nucleosome particle forming a ‘beads‐on‐a‐string’‐like arrangement along DNA is highly conserved across all species with very few exceptions (Soo & Warnecke, 2021). Bacterial chromatin is dominated by NAPs including HU, IHF, Hfq, and H‐NS (Dorman et al., 2020; Luijsterburg et al., 2008). Archaea likewise use a wide repertoire of NAPs including archaea‐specific proteins, proteins derived from horizontal gene transfer from bacteria like HU, and histones (Figure 1b). Intriguingly, some archaeal groups do not possess any genes encoding known NAPs in their genomes (Hocher et al., 2022).

Archaeal histones are structurally and in their sequence preference like eukaryotic histones, but they generally lack the N‐terminal tails that are key to the eukaryotic histone code and epigenetic regulation. We here refer to histones as the classic ‘nucleosomal’ histones, but are aware of several unusual histone variants in Archaea and Bacteria without characterised NAP function (Schwab et al., 2024). Histones are phylogenetically widely distributed in archaea and considered the prevalent chromatin proteins in euryarchaeota. The base units of the archaeal histones are dimers, which in the presence of DNA oligomerise to form a helical ramp with DNA wrapped around the protein core like a solenoid, but apparently without clearly defined ends and thus theoretically forming ‘endless’ particles referred to as ‘hypernucleosomes’. Each histone dimer wraps 30 bp of DNA and the dimensions including the diameter of the solenoid and the distance between DNA double strands for each turn are identical to the eukaryotic nucleosome particle (Henneman et al., 2018; Maruyama et al., 2013; Mattiroli et al., 2017). Current models suggest a highly dynamic ensemble where histone dimers continuously associate and dissociate from both ends of the hypernucleosome fibre. Histones are also widespread in ASGARD archaea but remain uncharacterised (Hocher & Warnecke, 2024). Crenarchaeota (recently reclassified as Thermoproteota) within the TACK superphylum of archaea are somewhat of an outlier as most members lack histones but rather employ a plethora of other NAPs such as Alba, Cren7 and Sul7, some of which are specific to crenarchaeotes or subgroups within (Hocher et al., 2022; Figure 1b).

## 3 ARCHAEAL GENE REGULATION—AN INCENTIVE FOR DEEPER STUDIES

The archaeal transcription machinery operating in this chromatin context is an ancestral version of eukaryotic systems, this includes homologues of RNAPII subunits (Rpo1‐13), general transcription factors enabling transcription initiation (TBP, TFB and TFE), transcription elongation (Spt4/5, Elf1 and TFS) and termination (aCPSF1, aka FttA); this is radically different from the bacterial transcription apparatus (Blombach et al., 2019; Fouqueau et al., 2018; Werner & Grohmann, 2011). The conserved nature of the transcription machinery and histones contrasts with the highly complex mechanisms of gene regulation in eukaryotes including coactivators/mediators, sophisticated epigenetic control of chromatin, all of which are apparently absent, or have not been discovered yet, in archaea. This pairing makes the interplay between chromatin and transcription in archaea a fascinating, yet understudied field of research. Our knowledge is mostly limited to Crenarchaeota and Euryarchaeota that are populated by well‐established model organisms. We follow on from previous reviews (Peeters et al., 2015; Sanders, Marshall, & Santangelo, 2019) and focus on recent data providing new insight into the chromatin landscape of archaeal chromosomes around promoters and their interconnection with gene expression. In addition, we review how these data compare to data testing the effect of chromatinisation on transcription in reconstituted systems.

## 4 THE ‘CHICKEN‐OR‐EGG’ PROBLEM OF CHROMATIN‐FREE PROMOTER REGIONS

Which came first, the chicken or the egg? Do high levels of transcription counteract chromatin condensation, or do high levels of chromatinisation counteract transcription? Keeping in mind the mutual interaction between transcription and chromatin, correlations between (i) transcription initiation factor binding to promoters (by ChIP‐seq), (ii) mRNA levels as proxy for promoter activity (RNA‐seq), and (iii) the chromatinisation state of the promoter region and the transcription unit (MNase‐seq) provide rich data for studying the association of chromatin with promoter activity in vivo. The last years have seen several studies mapping the chromatin landscape using MNase‐seq, a technique that relies on endo‐ and exonucleolytic digest of chromatin preparations by micrococcal nuclease (MNase) and subsequent isolation and identification of DNA sequences and regions protected from digestion (by chromatin) using high‐throughput sequencing (Ammar et al., 2012; Hocher et al., 2019; Maruyama et al., 2013; Nalabothula et al., 2013; Ofer et al., 2023). For archaea with histone‐chromatinised DNA, MNase‐seq data show a peak pattern of 30 bp multimers in the DNA fragment size distribution that corresponds to hypernucleosome particles of increasing sizes. It is important to keep in mind that many NAPs do not provide protection against MNase, possibly due to more labile binding, and would be ‘dark matter’ in these analyses. For example, *Pyrobaculum calidifontis* and *Saccharolobus solfataricus* are two crenarchaeotes that lack histones but use other NAPs such as Cren7. MNase digestion of chromatin preparations from these two species did not yield any specific DNA protection patterns (Maruyama et al., 2020), while *Thermoplasma acidophilum*, also lacking histones, showed a distinct digestion pattern of ~50 and ~85 bp fragments generated by the HU‐family protein HTa (Hocher et al., 2019). Several groups have applied MNase‐seq to show the formation of ‘chromatin‐free regions’ (CFR) by histones and HTa in different euryarchaeota (Ammar et al., 2012; Hocher et al., 2019; Maruyama et al., 2013; Nalabothula et al., 2013; Ofer et al., 2023). In a truly *Frankensteinian* experiment, the Warnecke team showed that the heterologous expression of the archaeal histone HMfA in *E. coli* forms CFRs around promoters on the *E. coli* chromosome (Rojec et al., 2019), which is likely due to the fact that both archaea and bacteria include A/T‐rich motifs in their promoter regions that disfavour DNA binding required for hypernucleosome formation. In line with this notion, in vitro reconstitution of *Thermococcus kodakaraensis* chromatin recapitulates the nucleosome‐sequencing pattern and CFR formation around promoters, suggesting that CFRs can form independently of active ongoing transcription or archaeal remodelling factors (Nalabothula et al., 2013).

**FIGURE 2:**
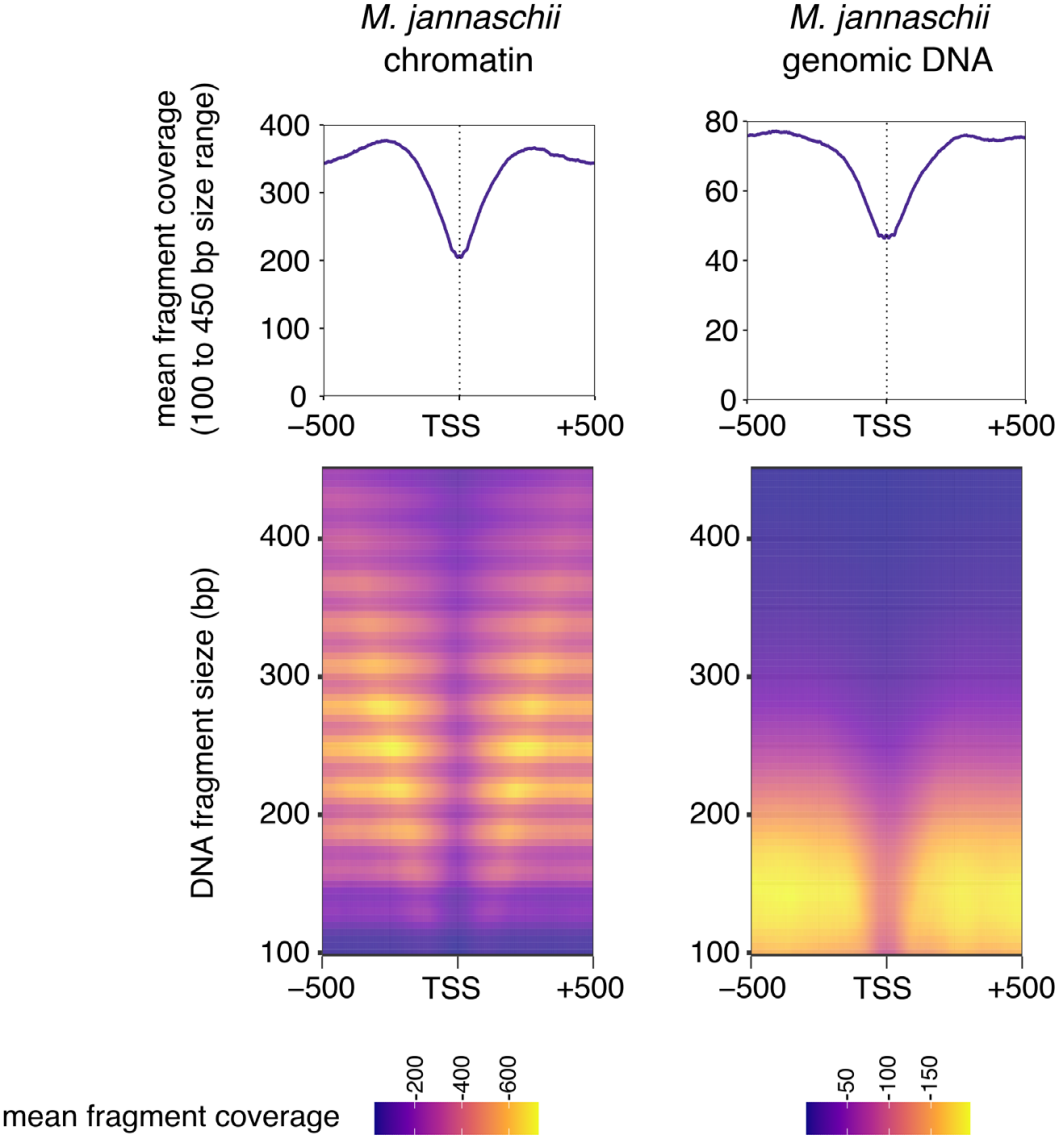
The problem of getting quantitative information on CFR formation. MNase‐seq data of *Methanocaldococcus jannaschii* chromatin preparation are strongly dominated by hypernucleosome particles yielding 30 bp multimer DNA fragments that form CFRs around TSSs. DNA coverage heatmaps were sorted by DNA fragment size and aggregated over 939 TSSs. Controls with dechromatinised genomic DNA digested by MNase under identical conditions show a radically different size distribution void of the 30 bp multimers, but the aggregate signal shows a similar relative decrease in DNA fragment coverage across TSSs attributed to the sequence bias of MNase. Despite the low background signal in the chromatin MNase‐seq data, it is therefore difficult to quantitatively assess CFR formation on individual promoters. Data were obtained from NCBI GEO GSE216101 (Ofer et al., 2023).

A considerable technical issue of MNase‐seq arises from the sequence bias of MNase which preferentially cleaves AT‐rich DNA—including promoter regions. This bias complicates the interpretation of MNase‐seq data (Chung et al., 2010). Control experiments where naked genomic DNA is subjected to MNase digestion show a fragment size distribution lacking completely the 30 bp multimer peaks in the DNA fragment size distribution typical for hypernucleosomes. However, these control experiments show a ‘dip’ in the aggregate signal around promoters that superficially appears very similar to MNase‐digested chromatin data (Figure 2). In theory, normalisation against such a control experiment with naked genomic DNA should remove the sequence bias of MNase (Ofer et al., 2023; Rojec et al., 2019). In practice, however, it is not possible to achieve a perfect control experiment. Digesting unchromatinised genomic DNA under the same experimental conditions yields a different DNA fragment size distribution that creates bias during the sequencing library preparation where the process involves the depletion of DNA fragments outside minimum and maximum length thresholds that affects these samples differently. Alternatively, reducing either the enzyme concentrations or the digestion time to obtain a similar DNA fragment size distribution means that the experimental conditions are not fully comparable. Therefore, a proper normalisation of MNase‐seq data is very challenging. Any quantitative assessment of the chromatinisation state of promoter regions versus promoter activity suffers from the MNase bias. With this note of caution in mind, a comparison between promoters belonging to the highest and lowest quintiles transcription levels in *Methanocaldococcus jannaschii* showed that, while CFRs are maintained regardless of actively ongoing transcription, there are qualitative differences between the promoters in the quasi‐normalised MNase‐seq signal (Ofer et al., 2023). In absence of a good data normalisation, it is the 30 bp multimer pattern in the DNA fragment size distribution that provides strong evidence that protection of DNA by hypernucleosomes shapes MNase‐seq data much more than MNase bias and hence CFRs are a real phenomenon. This important aspect is lost if one calculates the aggregate signal across all DNA fragment sizes.

## 5 NEW TRICKS FOR AN OLD DOG, ATAC‐SEQ AND NoMe‐SEQ

Modifications of MNase‐seq and alternative techniques have been developed to overcome its limitations. Conducting MNase‐seq with a range of MNase concentrations can reveal more labile NAP‐DNA interactions such as ‘fragile nucleosomes’ in eukaryotes (e.g. Mieczkowski et al., 2016). A different approach is to use chemical DNA cleavage reagents tethered to histones ex vivo rather than a nuclease (e.g. Chereji et al., 2018).

Two alternative methods to map chromatin occupancy have been now applied to archaea providing independent evidence for the formation of CFRs. Georgi Marinov and colleagues applied, ATAC‐seq and NoMe‐seq to the euryarchaeon *Haloferax volcanii* (Marinov et al., 2023). Standard MNase‐seq data already suggested the formation of CFRs in *H. volcanii* which was attributed to the single histone HstA (Ammar et al., 2012). Notably, agarose gel electrophoresis of MNase‐digested chromatin did not show the ladder pattern typical for hypernucleosome‐forming histones first discovered by Maruyama et al. in *T. kodakaraensis* (Maruyama et al., 2011, 2013). Subsequent proteome analyses (Dulmage et al., 2015) and molecular characterisation of HstA and its orthologue HypA in *Halobacterium salinarum* showed relatively low abundance and localised binding patterns on the chromosome for both histones incompatible with the function of HstA/HypA as major chromatin proteins (Sakrikar et al., 2023; Sakrikar & Schmid, 2021).

ATAC‐seq and NoMe‐seq results now provide a first quantitative view of CFR formation around promoters in *H. volcanii*. ATAC‐seq assesses the chromatin state through mapping chromosome regions accessible for tagging using hyperactive Tn5 transposase in chromatin preps in vitro (Buenrostro et al., 2013). NoMe‐seq is providing a measure of absolute chromatin occupancy; chromatin preparations are subjected to DNA methylation which is mapped by high‐throughput sequencing, and any protection against methylation reflects the accessibility of DNA that is inversely related to chromatinisation (Kelly et al., 2012). In addition, NoMe‐seq reports on the chromatinisation of longer DNA fragments reflecting the binding of multiple NAPs on the same DNA molecule and can thereby identify different states of chromatinisation possibly linked to a heterogenous cell population, although *H. volcanii* chromosomes appear overall to be in a similar chromatinisation state under exponential growth conditions (Marinov et al., 2023).

The *Haloferax* ATAC‐seq data are striking as they show no correlation between promoter accessibility and gene expression—*Haloferax* promoters seem to be in an accessible ground state (Marinov et al., 2023). If so, chromatinisation in Archaea might serve to shape the search space for basal transcription factors and regulators to promoters, somewhat similar to eukaryotic chromatin that limits the search space for transcription factors to nucleosome‐free regions (Ferrie et al., 2022).

**FIGURE 3:**
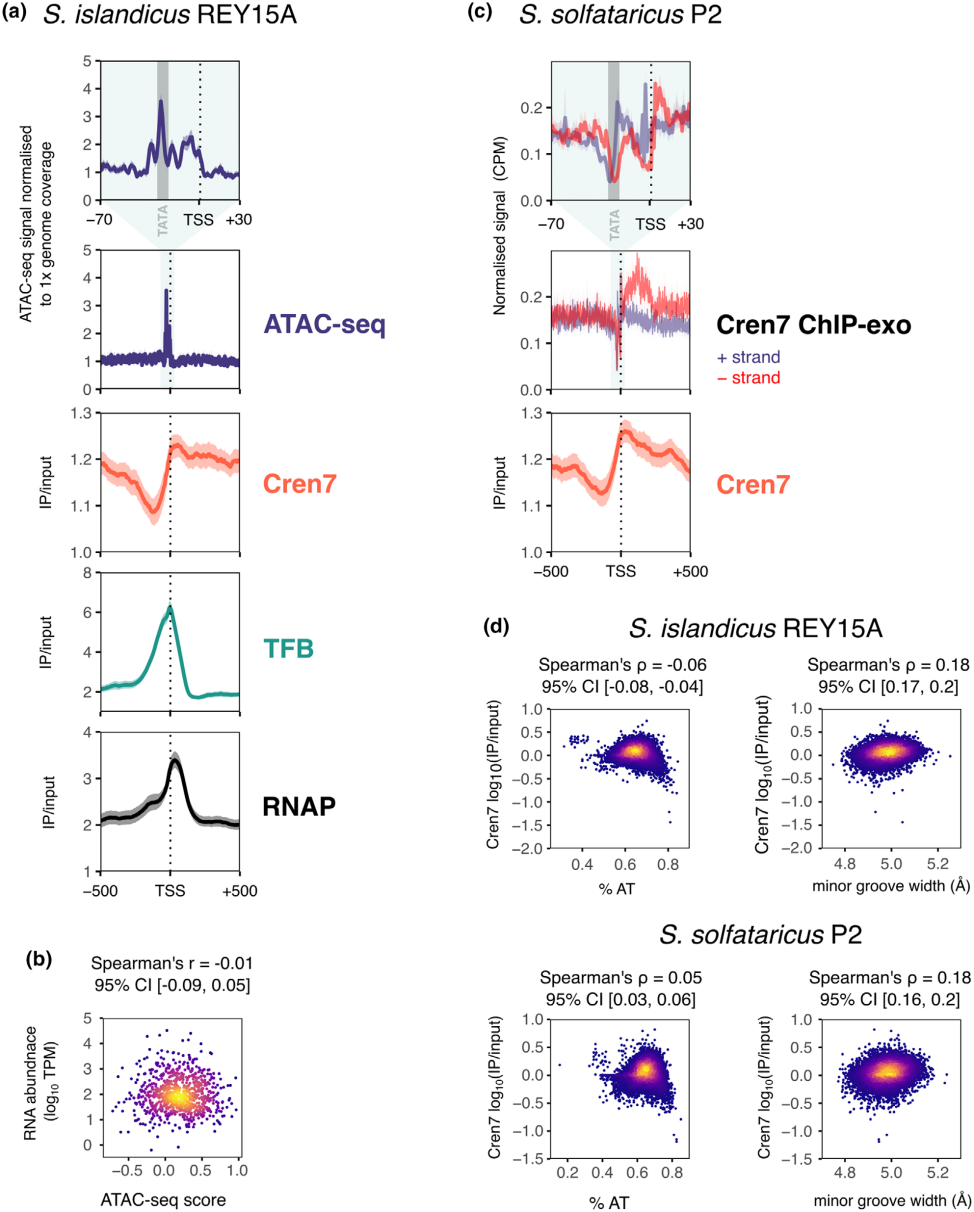
*Saccharolobus* chromatin forms narrow two‐partite CFRs around core promoter elements flanked by Cren7. (a) Aggregate plots for ATAC‐seq (NCBI SRA PRJNA814106; Badel et al., 2022) and Cren7, TFB and RNAP (Rpo4/7 subunits) ChIP‐seq signal (NCBI GEO GSE226026; Blombach et al., 2024) for 837 primary TSSs of coding genes in *Saccharolobus islandicus* REY15A. The line represents the mean (IP/input with SES scaling), the shaded area represents the 95% confidence interval calculated from 1000 bootstrap replicates. A representative of two biological replicates is shown for all data sets. On top, a zoom over 100 bp for the ATAC‐seq signal is shown along the standard position of TATA‐boxes. (b) Aggregate plots for Cren7 ChIP‐exo and ChIP‐seq data for 967 primary TSSs in *Saccharolobus solfataricus* P2 (Blombach et al., 2024). The line represents the mean (IP/input with SES scaling), the shaded area represents the 95% confidence interval calculated from 1000 bootstrap replicates. ChIP‐exo data represent the geometric mean of two biological replicates. For Cren7 ChIP‐seq, a representative of two biological replicates is shown. (c) ATAC‐seq signal is not correlated to gene expression. Scatter plot depicting the ATAC‐seq signal (NCBI SRA PRJNA814106; Badel et al., 2022) normalised to local background, see Section 12) versus transcript level estimates from RNA‐seq (NCBI GSE128063; Takemata et al., 2019) for 834 primary TSSs and their corresponding first cistron transcript levels. Spearman's ρ and the 95% confidence interval (Bootstrap) are shown on top. (d) ChIP‐seq data for Cren7 in *S. solfataricus* P2 and *S. islandicus* REY15A (NCBI GEO GSE226026) show no in vivo binding preference for AT‐rich DNA. Input‐normalised *S. islandicus* Cren7 ChIP‐seq occupancy was averaged over consecutive 200 bp bins with bins within 250 bp of CRISPR arrays omitted to remove the Cbp1‐mediated Cren7 DNA binding signal. Minor groove width was predicted using DNAShapeR (Chiu et al., 2016).

The NoMe‐seq data for *H. volcanii* reveal an overall high degree of chromatinisation (Marinov et al., 2023), which is enigmatic considering that homologues of known archaeal NAPs are only present in low abundance in *H. volcaniii* proteome (Hocher et al., 2022) and which hints at the importance of hitherto unknown NAPs in haloarchaeal chromosomes. ATAC‐seq data also provided the first quantitative glimpse at chromatin occupancy in crenarchaeotes. Marinov and colleagues re‐analysed ATAC‐seq data for *Saccharolobus islandicus* REY15A (Badel et al., 2022) and showed CFRs around promoter regions covering all the core promoter elements BRE, TATA and the region upstream of the transcription start site (TSS; Figure 3a). Thus, CFRs also form in archaeal species not encoding histones or HU homologues (Marinov et al., 2023). The extent of CFR formation appeared to be stronger in *H. volcanii* than in *S. islandicus* (Marinov et al., 2023). This could reflect biological differences where chromatin occupancy is more drastically reduced at *H. volcanii* promoters compared to those of *S. islandicus*. However, CFRs were only detectable in ATAC‐seq data after formaldehyde crosslinking in both *H. volcanii* and *S. islandicus*. Differences in the depth of CFRs might thus be influenced by differences in crosslinking efficiencies. CFR formation in *S. islandicus* should also be visible in ChIP‐seq data for individual NAPs. Because the *S. islandicus* CFRs are relatively short (~45 bp, Figure 3a) compared to the DNA fragment length distribution from a ChIP‐seq experiment, even a level of 100% CFR formation would be reflected in only a small decrease in ChIP‐seq signal. Indeed, there is a noticeable decrease in Cren7 ChIP‐seq occupancy at promoters followed by higher occupancy downstream of the TSS (Figure 3a). As in *H. volcanii*, the ATAC‐seq signal on the CFRs shows no significant correlation with gene expression (Figure 3b).

**FIGURE 4:**
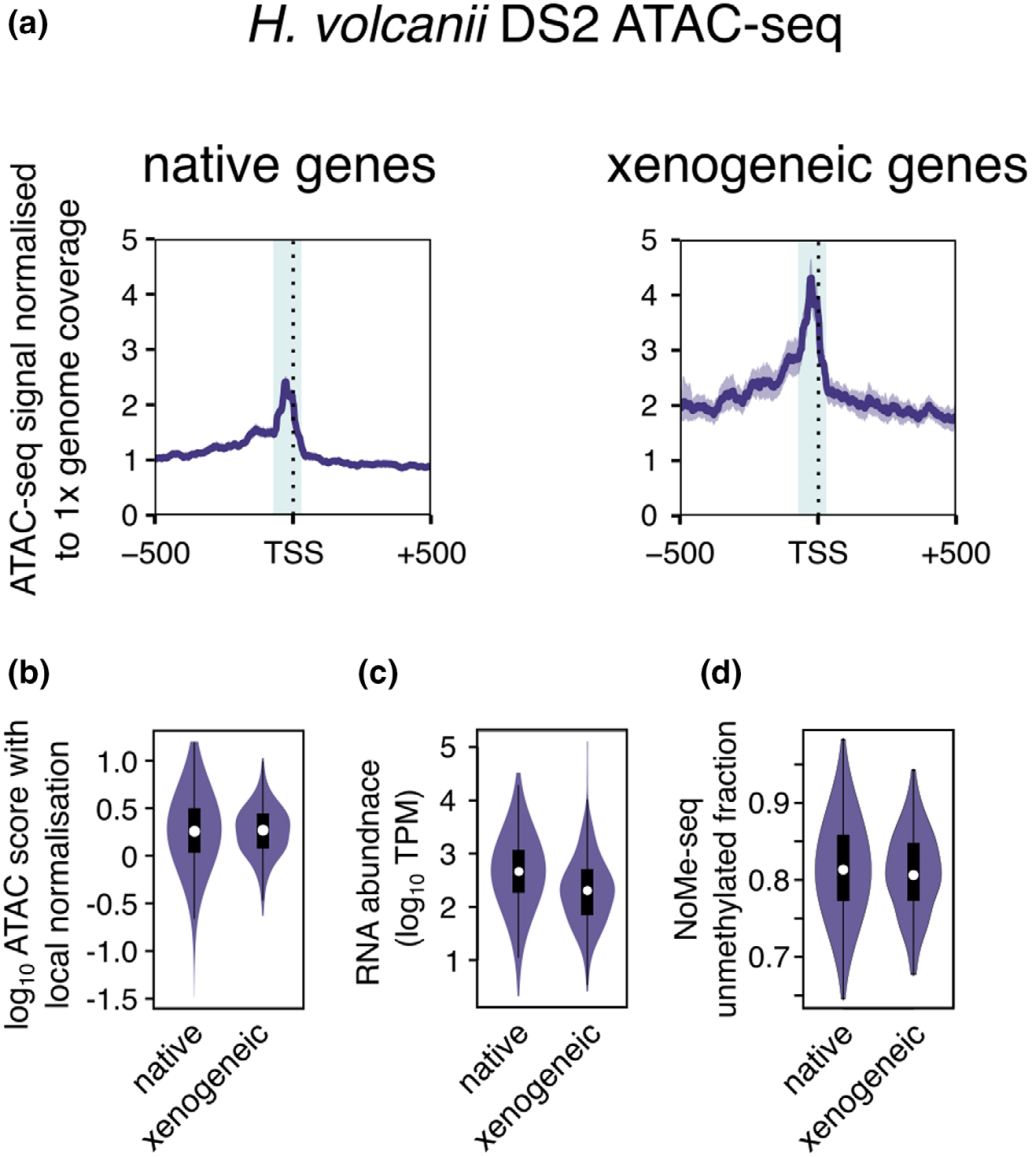
Promoters of xenogeneic genes reside within CFRs similar to native genes in *Haloferax volcanii*. Kmeans clustering was applied to the rare codon fraction of genes with mapped TSSs. (calculated according to Hartman et al., 2010) resulting in two clusters of native (*n* = 3364) and xenogeneic genes (*n* = 629). ATAC‐seq signal (NCBI GEO GSE207470; Marinov et al., 2023) for a 1000 bp interval around the mapped TSS of genes belonging to each cluster was calculated and intervals were filtered out to mitigate potential problems with mapping over repetitive sequences. Representative data for one of two biological replicates are shown. TSS mapping data were obtained from (Babski et al., 2016). (a) Aggregate plots showing the mean signal (scaled to counts per million, cpm) for one representative biological replicate out of two. The shaded area represents the 95% confidence interval calculated from 1000 bootstrap replicates. The shaded area represents the same −40 to +5 interval (relative to the TSS) as highlighted in Figure 3a to compare the width of the ATAC peak signal around promoters between *H. volcanii* and *Saccharolobus islandicus*. (b) Violin plots showing the quantification of the ATAC‐seq signal with local background subtracted. (c) Violin plots comparing transcript levels for the same set of native and xenogeneic genes as above. Transcript level estimates were obtained from (Blombach et al., 2018; NCBI GEO GSE101134). (d) Violin plots comparing absolute chromatinisation levels (1—NoMe‐seq signal) between the same sets of native and xenogeneic promoters.

ChIP‐exo is a ChIP‐seq derivative where resolution is improved by 5′–>3′ exonuclease digest of the immuno‐precipitated DNA fragments to map the borders of the protein‐DNA complexes at nucleotide resolution (Rhee & Pugh, 2011). Aggregate ChIP‐exo data for *S. solfataricus* P2 Cren7 show two regions of strong Cren7 depletion at promoters: the TATA‐box and immediately upstream of the TSS that are each flanked downstream by Cren7 binding sites (Figure 3c). The two Cren7‐depleted regions match the two local maxima in the *S. islandicus* aggregate ATAC‐seq signal (Figure 3a). These data suggest that CFRs in *Sulfolobales* might not constitute a region with uniformly lower chromatin occupancy but rather tolerate binding of NAPs like Cren7 at some positions. The promoters are likely to require clearing of these flanking NAPs to form a transcription pre‐initiation complex with RNAP and TFE across the TSS. Notably, the *Sulfolobales* CFRs appear considerably narrower than those in *H. volcanii* (Marinov et al., 2023), where the ATAC signal covers also the region upstream of the BRE/TATA‐box, including thus many more binding sites of transcription regulators (Figure 4a). This likely influences the way regulators have evolved and operate in the two archaeal groups.

In conclusion, cren‐ and euryarchaeota with very different NAP repertoires all contain CFRs that keep the promoter accessible to transcription factors and RNAP. Whether this holds true for all archaea remains to be seen. This is in sharp contrast to eukaryotes, which expend vast resources on chromatin remodelling factors that fine tune access of factors to regulatory elements including promoters and enhancers. Beside its possible role in guiding the search of transcription factors for promoters, the ordered chromatin landscape around archaeal promoters could regulate transcription in other ways than promoter occlusion.

## 6 DOES CHROMATINISATION AFFECT PROMOTER‐PROXIMAL TRANSCRIPTION TERMINATION?

RNA polymerase ChIP‐seq data from *S. solfataricus* and *S. islandicus* (Blombach et al., 2021; Blombach et al., 2024; Figure 3a) and KAS‐seq mapping of ssDNA regions that reflect single‐stranded DNA of transcription elongation complexes in *H. volcanii* (Marinov et al., 2023) demonstrate that RNAPs accumulate in promoter‐proximal regions in cren‐ and euryarchaeotes. ChIP‐seq data from *S. solfataricus* indicate that the promoter‐proximal RNAP accumulation includes complete transcription elongation complexes encompassing RNAP‐associated elongation factors Spt4/5 and Elf1. The level to which the promoter‐proximal transcription elongation complexes escape downstream into the body of the transcription unit correlates strongly with mRNA levels genome‐wide (Blombach et al., 2021). The escape level is dynamic and fine tunes transcription in response to changes in the environment including stress. It is a bona fide mechanism of global gene regulation. The underlying molecular mechanism involves the archaeal termination factor aCPSF1, aka FttA, which according to ChIP‐seq data is associated with promoter‐proximal transcription elongation complexes in a fashion that is anticorrelated with mRNA levels. In other words, transcription appears to be widely regulated by premature termination in *S. solfataricus* and, as all the components are highly conserved, likely also in other archaea (Blombach et al., 2021). aCPSF1 has two distinct paralogs in metazoans, CSPF73 that is part of the large CPSF complex which facilitates transcription termination at the end of coding genes (Rodriguez‐Molina et al., 2023), and Int11, the catalytic subunit of the Integrator complex in metazoans which facilitates premature termination of promoter‐proximally stalled RNAPII (Wagner et al., 2023). Thus, in a fascinating case of convergent evolution, a single protein present in all extant archaea evolved to function in termination and transcription regulation via premature termination in archaea and metazoans.

But how are promoter‐proximal transcription dynamics and aCPSF1‐mediated premature transcription termination mechanistically regulated? The chromatin environment could play a role in establishing the promoter‐proximal dynamics of transcription. As proof of principle, the combination of histones mutant variants with increased DNA affinity with a DNA binding site itself optimised for high affinity using SELEX (Bailey et al., 2000) leads to substantial pausing of RNA polymerases in front of the hypernucleosome particle (Wenck et al., 2024). These effects may be weaker or stronger in vivo with wild‐type NAPs and binding sites, but it is conceivable that hypernucleosome particles positioned downstream of the promoter could shape promoter‐proximal elongation dynamics somewhat akin for example to RNA polymerase accumulation upstream of the +1 nucleosome in yeast during heat shock response (Vinayachandran et al., 2018). A crucial question to this end is whether WT histones and other archaeal NAPs such as those in *H. volcanii* and Sulfolobales can elicit similar transcriptional pausing effects. On the other hand, the increased residence time of RNA polymerase and transcription initiation and elongation factors at the promoters of many weakly transcribed genes will compete with NAP binding and therefore help to maintain CFRs.

## 7 RECONCILING IN VITRO TRANSCRIPTION AND FUNCTIONAL GENOMICS

While correlations between genome‐wide NAP profiling and transcriptome data inform about associations between NAP binding and mRNA levels, in vitro transcription experiments are crucial to establish a causal relationship between DNA chromatinisation, promoter access of initiation factors and transcription elongation dynamics.

In experiments where histones are allowed to compete with the transcription machinery for access to the promoter in vitro, they exert a strong repressive effect on transcription (Ofer et al., 2023; Wilkinson et al., 2010), and this also holds true for the unusual tetrameric histone variant MJ1647 (Ofer et al., 2023). In agreement, the transcription regulator Ptr2 that activates transcription by enhancing the recruitment of the initiation factor TBP to the promoter's TATA‐box (Ouhammouch et al., 2004) counteracts repression by canonical histones in *M. jannaschii* (Wilkinson et al., 2010). These results suggesting that histone‐chromatinisation of promoters is an integral part of transcription regulation are at odds with the apparent sequence‐driven, transcription‐independent formation of CFRs around promoters in *M. jannaschii* in vivo (Ofer et al., 2023). The relatively short, linear DNA templates used in in vitro transcription experiments may not fully recapitulate CFR formation and the experiments might not reflect the histone to DNA ratios present in vivo. Despite these discrepancies and caveats, the experiments show the potential of histones to regulate access to promoters. Another caveat of the in vitro assays is the crucial choice of a representative promoter and native transcribed sequence. Synthetic transcription templates with SELEX‐optimised binding sites have been subsequently shown to form high‐affinity complexes with histone tetramers that differ from hypernucleosomes in their DNA compaction properties (Erkelens et al., 2023). Archaeal histones can decrease the transcription elongation rate, defined as nucleotides synthesised per second, and, theoretically also decrease transcription processivity, defined as average nucleotides synthesised per initiation event—but histones do not block transcription completely (Ofer et al., 2023; Sanders, Lammers, et al., 2019; Wenck et al., 2024; Xie & Reeve, 2004). A recent, more detailed investigation testing the effect of HTkA from *T. kodakaraensis* binding to templates encoding a high‐affinity histone binding site showed a ~20% decrease in the elongation rate (Wenck et al., 2024). While pausing and a slower elongation rate per se do not affect transcription output under steady‐state conditions, altered transcription elongation properties could make a difference if they affect downstream processes such as premature transcription termination or coupled translation of the nascent mRNA.

Beyond archaeal histones, little is known about the impact of NAPs on transcription. In multi‐round transcription assays that cannot discriminate between effects on transcription elongation and initiation, *S. solfataricus* Alba efficiently repressed transcription whereas Sul7d did not show any significant effect (Bell et al., 2002). Like the euryarchaeal histone variant MJ1647, Alba can bridge DNA molecules (Laurens et al., 2012), but whether the repressive effect of Alba is linked to its ability to interlink regions of the chromosome, and potentially trap RNA polymerases in the loops formed, is unclear. Alba can heterodimerise with its paralog Alba2, which limits the formation of longer filaments but any impact on transcription still remains to be explored (Laurens et al., 2012). The pleiotropic effects on transcription by Cren7 and the CRISPR array‐binding Cbp1 NAPs are discussed further below.

## 8 DO ARCHAEA FORM HETEROCHROMATIN‐LIKE DOMAINS TO SILENCE XENOGENEIC GENES?

While there is little evidence for NAPs mediating extensive regulation of promoter access, a different question is whether there are specific regions in archaeal chromosomes that are silenced by chromatinisation. In bacteria including *E. coli* and *B. subtilis*, NAPs such as Fis, Hfq (in its role as NAP besides its RNA chaperone function) and H‐NS mediate the transcriptional silencing of genomic regions enriched in xenogeneic genes, i.e. genes incorporated into the chromosome by lateral gene transfer including DNA conjugation, natural plasmid transformation and bacteriophage or virus integration (Amemiya et al., 2021). The reasons why xenogeneic DNA often has a higher AT content compared to cellular chromosomes is still a matter of debate, but the AT preference of NAPs including Fis, H‐NS, and Hfq is striking and could play an instrumental role in this bias, as silenced foreign genes are better tolerated and more likely to be retained (Beaufay et al., 2021; Duan et al., 2021). This type of bacterial transcriptional silencing is reminiscent of heterochromatin formation in eukaryotes.

Even though the transcription initiation factors of archaea and bacteria are most likely not evolutionary related (Fouqueau et al., 2017), the promoter motifs are AT‐rich in both domains. The archaeal TATA‐box (5′‐TTTATATA‐3′ in *M. jannaschii*) interacts with TBP and the bacterial ‘−10’ element (5′‐TATAAT‐3′ in *E. coli*) is recognised by sigma factor, respectively. This leads to the surprising finding that archaeal genome fragments can often be transcribed in bacteria (e.g. Fiorentino et al., 2009). Moreover, AT‐rich non‐promoter sequences from xenogeneic DNA may be recognised by the host and generate RNA transcripts that negatively interfere with the gene expressions program. Therefore, the selection pressure to silence xenogeneic genes likely applies to archaea as it does in bacteria.

Many archaeal genomes are populated with highly abundant mobile genetic elements (Brugger et al., 2002; Krupovic et al., 2019; Makarova et al., 2014; Wu et al., 2022). For example, the euryarchaeon *H. volcanii* DS2 has seven AT‐rich genomic regions containing insertion sequence (IS) elements and a putative prophage (Hartman et al., 2010). Xenogeneic genes can be easily identified in *Haloferax* based on codon usage and AT content (Hartman et al., 2010), and transcriptome analyses of transcription factor knockout strains indicate that the promoters of xenogeneic genes depend to a larger degree on the basal factor TFE compared to native genes (Blombach et al., 2018). The reason for the greater TFE dependence for transcription initiation could be a different chromatin environment in xenogeneic genes. However, plotting the ATAC‐seq signal (Marinov et al., 2023) comparing native and xenogeneic genes shows that the promoters of xenogeneic genes remain generally accessible (Figure 4a,b) which agrees with transcript level estimates that show them only slightly lower than those of native genes (Figure 4c). Xenogeneic genes show generally a higher baseline signal around the promoters which is likely due to AT‐bias in the ATAC‐seq signal (Marinov et al., 2023). Notably the corresponding NoMe‐seq signal is similar for both types of promoters (Figure 4d). These data do not support the silencing of xenogeneic genes in *H. volcanii*. In addition, Marinov also reported that no larger scale domains of increased chromatinisation appear to be present in *H. volcanii* (Marinov et al., 2023). Chromosome conformation capture experiments using Hi‐C reveal that the *Sulfolobus* genome is compartmentalised, i.e. separated into two compartments (A and B) that differ in terms of overall transcription activity. Mobile genetic elements are enriched in the transcriptionally less active B compartment of the chromosome (Badel et al., 2022; Takemata et al., 2019; Takemata & Bell, 2020). The B compartment shows overall lower ATAC‐seq signal suggesting higher chromatin occupancy levels (Badel et al., 2022). These data, however, aggregate over many genes with different transcription levels and do not necessarily indicate the existence of transcriptionally silenced, heterochromatin‐like regions. Our primary TSS mapping data for *S. islandicus* REY15A that we used for the CFR plots in Figure 3 included too few genes belonging to the ‘mobilome’ class in the arCOG database (Makarova et al., 2015) for a thorough analysis of xenogeneic promoters as in *Haloferax*, possibly because these gebes show too low transcription activity for TSS mapping.

A different way to identify possibly heterochromatin‐like regions is to test for increased occupancy of individual NAPs at specific regions of the genome, in particular AT‐rich regions.

Cren7 on its own has a binding preference for AT‐rich DNA in vitro (Zhang et al., 2019) but a re‐analysis of our recent ChIP‐seq data for Cren7 in *S. solfataricus and S. islandicus* does not provide any consistent evidence of increased Cren7 binding to genomic regions with higher local AT content (Figure 2d). Indeed, regions with the highest AT content tended to have low Cren7 occupancy. Instead, Cren7 occupancy correlated weakly but significantly with the predicted minor groove width (Figure 3d) in line with the DNA‐binding mode of Cren7 (Zhang et al., 2010). ChIP‐seq data for Sul12a from *S. acidocaldarius* and its *S. islandicus* homologue aDCP1 (Lemmens et al., 2022; Zhang et al., 2023) were reported to not show any strong local enrichment.

## 9 DUAL‐FUNCTION CHROMATIN IN ARCHAEA

The boundary between regulatory transcription factors and NAPs is not sharp, but rather a continuum, as some transcription factors have the ability to polymerise along the DNA leading to the formation of chromatin‐like structures (at least in vitro; e.g. Peixeiro et al., 2013), while many NAPs show some level of sequence‐specificity. Arguably on the outer edges of this spectrum lies Cbp1, a protein that binds with nanomolar affinity to repeat sequences mainly found in the very long (in the 10 kb range) CRISPR arrays but also in other locations of the genomes of *S. islandicus* and *S. solfataricus* (Blombach et al., 2024; Peng et al., 2003). Cbp1 physically interacts with the generic crenarchaeal NAP Cren7 in a defined 1:2 stoichiometry via the third helix‐turn‐helix domain of Cbp1 in a DNA‐dependent manner; both factors together form a highly regular chimeric chromatin structure in vivo (Blombach et al., 2024). Cbp1 has pleiotropic effects on transcription; on one hand, it is a positive elongation factor as Cbp1 enhances the synthesis of the long nascent CRISPR RNAs directed by the leader promoters of the arrays, and on the other it represses the activity of cryptic promoters that were incorporated into the arrays by random spacer acquisition from xenogeneic DNA, typically viral or plasmid sequences. Cbp1 binding to repeat‐like sequences outside of CRISPR arrays inhibits the transcription from promoters overlapping with, or proximal to these binding sites. Intriguingly, the deletion of *cbp1* in *S. islandicus* REY15A widely affects gene expression in a region within one chromatin interaction domain (CID) that encompasses the two CRISPR arrays now lacking Cbp1‐Cren7 chromatinisation. This supports the notion that a CID compartment somehow physically encloses or isolates a subset of genes that are actively coregulated.

Members of the IS110‐family transposon in *S. solfataricus* and *S. islandicus* harbour two conserved Cbp1 binding sites, which are unusual in as much as they are not co‐opting Cren7 (Blombach et al., 2024). The deletion of Cbp1 leads to the activation of a cryptic promoter inside the IS110 transposons, indicating that Cbp1 somehow keeps the transposons in check, possibly by restricting transposition events. However, the molecular details of this regulation have not been investigated yet.

## 10 HETEROGENOUS CHROMATIN COMPOSITION

How do NAPs bind the chromosome in competition with other NAPs in the crowded environment of the archaeal cell? How do those NAPs interact with each other and are they able to form locally different chromatinisation states that could potentially regulate transcription?

Histone paralogues provide an obvious starting point to investigate mixed oligomeric chromatin structures in archaea. Most archaea encode two or more histone paralogues. The most likely scenario is that distinct histone paralogues do not populate distinct regions of the genome, but rather form mixed heterodimers which further polymerise in the presence of DNA, although direct experimental evidence is still lacking (Stevens et al., 2020). While the substantial differences between H2A, H2B, H3 and H4 histones enable the formation of a distinct octameric nucleosome particle in eukaryotes, structural models of archaeal nucleosomes are fibres without ‘ends’. However, this is not necessarily the case in vivo, where the incorporation of ‘capstone’ histone variants could form an end by disfavouring the growth of the hypernucleosome particles, possibly in a single direction (Stevens et al., 2020). The non‐canonical histone variant MJ1647 is unable to dimerise with canonical *M. janaschii* histones (like A1, A2 and A3) when co‐expressed in *E. coli* (Ofer et al., 2023). The 27 amino acid C‐terminal extension (Li et al., 2000) forms a ‘tetramerization module’ (Ofer et al., 2023). It is possible that this C‐terminal extension allowed for the speciation/evolution of an MJ1647‐specific homodimer interface, analogous to histone fusion genes that were proposed to drive the evolution of the heteromeric nucleosome complex (Irwin & Richards, 2024). Hypernucleosomes formed by canonical dimeric histones and tetrameric MJ1647 are likely to form separate filaments in *M. jannaschii*. However, it remains unclear to which extent MJ1647 binds to specific regions in the chromosome.

Proper differential chromatinisation in a histone‐containing archaeon has been demonstrated in *T. kodakaraensis* where TrmBL2 and Alba form chromatin structures that are distinct from hypernucleosome‐chromatin (Maruyama et al., 2011). The TrmBL2‐enriched chromosomal regions are relatively short and the binding of TrmBL2 to promoters is associated with transcriptional repression (Maruyama et al., 2011). The basis for the differential recruitment of TrmBL2 to these sites is poorly understood.

One way to generate such differential recruitment to specific DNA regions in absence of DNA binding specificity is to go partly ‘piggyback’: The example of the chimeric Cbp1‐Cren7 complex shows that low‐affinity binding NAPs like Cren7 may use protein–protein interactions with a pre‐bound sequence‐specific NAP like Cbp1 to increase their affinity for the DNA template leading to locally enhanced Cren7 occupancy (Blombach et al., 2024).

## 11 OUTLOOK

Improved deep sequencing techniques have generated genome‐wide binding profiles of many NAPs, transcription factors and RNAP at single nucleotide resolution which provide insights into the connection between chromatin organisation and the transcription apparatus in unprecedented detail. Combined with the emerging knowledge about the molecular structure of the components, the 3‐dimensional organisation of the chromosome, and sophisticated methods to test the impact of chromatin on transcription in vitro, as well as assessing associations between chromatin and transcriptome in vivo, allow us to address the big questions about the complex interplay between the genome and gene expression in archaea. Future lines of enquiry include:

1. A focus on *mutual causality*—how does transcription shape the chromatin landscape and how does chromatin regulate transcription?
2. An *appreciation of the diversity* of chromatin in different archaea—what are the selection pressures that shape chromatin and gene regulation?
3. How come that different NAPs all seem to form CFR? Are CFRs an ancestral trait that new NAPs thus evolve to maintain? Or did CFR formation evolve independently anew by convergent evolution with possibly different roles in transcription regulation?
4. Direct visualisation of the nucleoid and chromatin in cells by cryo‐Electron Tomography.

## 12 METHODS

### 12.1 *Methanocaldococcus jannaschii* MNase‐seq heatmaps

Sequencing data were obtained from GEO GSE216101 and aligned to the *M. jannaschii* genome as described in (Ofer et al., 2023). Raw DNA fragment coverage was calculated using the bamCoverage function (deeptools 3.1.3) for fragments within a size range of consecutive 5 bp windows from 100 to 450 bp. Coverage data for 1000 bp intervals around 939 mapped TSSs (Smollett et al., 2017) were imported into the R 4.2.1 environment using rtracklayer 1.58.0 and visualised using ggplot2 3.5.1.

### 12.2 *Haloferax volcanii* ATAC‐seq aggregate plots

Native and xenogeneic genes were split by kmeans clustering based on their rare tRNA score using data from (Blombach et al., 2018). Processed ATAC‐seq data were obtained from NCBI GSE 207470 (Marinov et al., 2023). Coverage data were calculated for the subset of genes with mapped TSSs (Babski et al., 2016) using deeptools 3.1.3 computeMatrix for a 1000 bp interval. Data were imported into R 4.2.1 and intervals with possible mappability issues resulting in low coverage (defined here as 0 coverage within 50 bp windows of each interval) were removed. Coverage data for the resulting intervals for 820 native TSSs and 167 xenogeneic genes were used to produce aggregate plots with ggplot 3.5.1. To calculate the normalised ATAC‐seq score, coverage of a window −40 to +5 relative to the TSS was divided by the average of 45 bp windows shifted 200 bp upstream and downstream. Mean absolute chromatin occupancy based on NoMe‐seq data (NCBI GSE 207470 as well; Marinov et al., 2023), was calculated for the same two 45 bp windows flanking the CFR 200 bp upstream and downstream.

### 12.3 *Saccharolobus islandicus* ATAC‐seq and ChIP‐seq aggregate plots and correlation with RNA‐seq

ATAC‐seq sequencing data were obtained from NCBI SRA PRJNA814106 and processed as described in (Badel et al., 2022). Processed Cren7, TFB, and Rpo4/7 ChIP‐seq data were obtained from NCBI GEO GSE226026 (Blombach et al., 2024). Coverage data were calculated for 1000 bp intervals around primary TSSs (Blombach et al., 2024) using deeptools 3.1.3 computeMatrix and intervals with any position with less than 20 reads coverage in the ChIP‐seq chromatin input data were removed. Data were imported into R 4.2.1 and aggregate plots were produced using ggplot2 3.5.1. ATAC‐seq scores were calculated as described above and correlated with published RNA‐seq data (Takemata et al., 2019).

### 12.4 *Saccharolobus solfataricus* Cren7 ChIP‐seq and ChIP‐exo aggregate plots

Data were obtained from NCBI GEO GSE226026 (Blombach et al., 2024) and plotted as described above using previously published TSS mapping data (Wurtzel et al., 2010).

## AUTHOR CONTRIBUTIONS

**Fabian Blombach:** Conceptualization; investigation; writing – original draft; visualization; software; writing – review and editing. **Finn Werner:** Funding acquisition; conceptualization; supervision; writing – review and editing.

## ETHICS STATEMENT

The authors have nothing to report.

## Data Availability

Data sharing not applicable to this article as no datasets were generated during the current study.
